# A clinical efficacy analysis of ankle arthrography-assisted modified ankle capsule repair in the treatment of recurrent bunion cysts

**DOI:** 10.3389/fsurg.2025.1553820

**Published:** 2025-05-07

**Authors:** Yongbin Fu, Siwei Mei, Mingming Ma, Xiaojun Ruan, Tao Ning

**Affiliations:** Department of Microsurgery and Reconstruction, Fuyang people’s Hospital Affiliated to Anhui Medical University, Fuyang, Anhui Province, China

**Keywords:** ankle, angiography, cyst, bunion, surgical outcomes

## Abstract

**Objective:**

This study aims to retrospectively analyze the clinical efficacy of ankle arthrography-assisted modified ankle capsular repair for treating recurrent bunion cysts.

**Methods:**

Clinical data from 16 cases of recurrent bunion cysts, treated between December 2021 and June 2024, were collected. All patients underwent intraoperative ankle arthrography with cyst excision, followed by ankle capsule repair based on the arthrography results. Patient gender, age, the presence of ankle arthrography during interstitial traffic with the cyst, and any postoperative complications were documented. Clinical outcomes were monitored during the postoperative follow-up.

**Results:**

In 14 out of 16 patients, ankle arthrography revealed communication of the contrast medium with the bunion cyst through the tendon sheath of the Flexor hallucis longus (FHL) tendon. In the remaining two patients, no contrast medium spillage was observed. The surgical incisions healed without infection, and a follow-up period of 3–24 months revealed only one recurrence.

**Conclusion:**

Ankle arthrography-assisted modified ankle capsule repair is an effective method to reduce recurrence rates in patients with recurrent bunion cysts, particularly those communicating with the ankle joint via the FHL tendon sheath.

## 1 Introduction

Cysts are commonly managed through methods such as manual compression, aspiration, or surgical excision. Although cysts of the great toe are relatively rare, their unclear pathogenesis often leads to high recurrence rates, prompting patients to seek repeated medical treatments. Literature reports that the recurrence rate following simple cyst excision can be as high as 62% (1–3), which not only imposes a physical burden on patients but also exacerbates their financial strain. In recent years, advancing research into the pathophysiology of great toe cysts has revealed a potential connection to the ankle joint. Notably, the use of ankle joint imaging techniques has been instrumental in clarifying this association. Imaging studies have shown that some great toe tenosynovial cysts are linked to the ankle joint through the flexor hallucis longus tendon sheath (4–6). This finding offers new insights for the surgical management of great toe cysts.

This study retrospectively analyzes the clinical data of patients with recurrent great toe cysts who underwent surgical treatment assisted by ankle joint imaging. By accurately localizing the cysts during surgery with imaging guidance and incorporating an improved ankle joint capsule repair technique, this study aims to evaluate the efficacy of this approach in reducing recurrence rates and improving clinical outcomes for patients. The results of this analysis are presented below.

## 2 Clinical data

### 2.1 General information

This study included clinical data from 16 patients with recurrent great toe cysts treated between December 2021 and June 2024. Among these patients, 12 were male (75%) and 4 were female (25%), with ages ranging from 26–72 years and an average age of 54.87 ± 11.67 years. Recurrence followed different initial treatments: five cases were recurrent after surgical excision, nine recurred after conservative treatments (including manual compression, spontaneous rupture, or aspiration), and two cases experienced recurrence after both surgical excision and conservative treatment, See Table 1. Regarding the affected side, three cases involved the left foot (18.8%) and 13 cases involved the right foot (81.2%). Detailed patient information is summarized in Table 2.

**Table 1:** Individual Follow-up Duration and Recurrence Status.

Case	Follow-up Duration (months)	Recurrence (Yes/No)
1	18	No
2	24	No
3	20	No
4	12	No
5	22	No
6	14	No
7	18	No
8	3	No
9	6	No
10	9	No
11	5	Yes
12	15	No
13	16	No
14	12	No
15	3	No
16	13	No

**Table 2:** General information of patients and intraoperative imaging results.

Case	Gender	Age (years)	Duration (months)	Side	Medical History	Ankle Joint Imaging (0: No, 1: Yes)	Communication with FHL Tendon Sheath	Reached Great Toe
1	Male	48	9	Right	Surgical excision × 1	1	1	1
2	Female	60	1	Right	Compression rupture × 1	1	1	1
3	Female	49	36	Right	Compression rupture × 1	1	1	1
4	Male	35	36	Right	Surgical excision × 1	1	1	1
5	Male	56	24	Left	Spontaneous rupture × 2	1	1	1
6	Female	60	18	Right	Surgical excision × 1, Aspiration × 1	1	1	1
7	Male	69	60	Right	Surgical excision × 2	1	1	1
8	Male	26	1	Left	Compression rupture × 1	1	1	1
9	Male	57	5	Left	Surgical excision × 2	1	1	1
10	Male	57	2	Left	Compression rupture × 1	1	1	1
11	Female	50	24	Left	Compression rupture × 1	0	0	0
12	Female	72	2	Right	Compression rupture × 1	1	1	1
13	Male	62	120	Left	Surgical excision × 3, Spontaneous rupture × 1	1	1	1
14	Male	53	2	Right	Spontaneous rupture × 1	1	1	1
15	Male	62	6	Right	Compression rupture × 1	0	0	0
16	Female	62	12	Left	Surgical excision × 1	1	1	1

Inclusion Criteria: All consecutive patients with recurrent great toe cysts presenting with pain and discomfort after prior surgical excision or conservative treatment were included. Recurrence was defined as the reappearance of a clinically detectable mass at the same location after prior treatment, confirmed by physical examination and ultrasound. All patients underwent preoperative MRI to evaluate the relationship between the cyst and surrounding anatomical structures.

**Exclusion Criteria:** First-time cases of great toe cysts and cysts attributable to known underlying medical conditions.

All patients were followed up for a period ranging from 3 to 24 months (mean 12.5 ± 6.7 months), with 12 patients (75%) having a follow-up period of 12 months or longer. Table 1 presents individual follow-up durations and recurrence status. Only one patient (Case 11) experienced recurrence at 5 months post-operation, which was one of the two cases where no communication between the ankle joint and the cyst was detected through arthrography.

### 2.2 Ankle joint imaging and surgical procedure

General anesthesia or epidural anesthesia was administered, and the patient was positioned supine. A pneumatic tourniquet was applied to the proximal thigh to control bleeding. A puncture was made at the anterior medial ankle joint space, followed by the injection of 20 ml of iodized contrast agent. The ankle joint was repeatedly flexed and extended, and after five minutes, fluoroscopic imaging was performed using a C-arm x-ray machine in the lateral view of the affected foot to detect any leakage of the contrast agent into the ankle joint. The relationship between the contrast medium, the flexor hallucis longus (FHL) tendon sheath, and the great toe cyst was also assessed.

For accurate anatomical localization, the puncture was made 1 cm medial to the anterior tibial tendon at the level of the ankle joint line. The contrast agent was injected under fluoroscopic guidance to ensure intra-articular placement. The ankle joint was then moved through a full range of motion (20 repetitions of plantar flexion and dorsiflexion) to facilitate contrast distribution.

If no leakage of the contrast agent was observed, an incision was made over the cyst, and dissection was carried out along the outer wall of the cyst down to its base. The cyst, along with its base and a portion of the normal tendon sheath, was completely excised, and the base was repaired as thoroughly as possible.

If contrast agent leakage was observed extending through the FHL tendon sheath to the great toe, additional surgical steps were taken. A longitudinal incision was made along the FHL tendon at the proximal first metatarsal, exposing and excising approximately 5 cm of the tendon sheath, with the excised tissue preserved for later use.

The longitudinal incision was made through the skin and subcutaneous tissue, extending 5 cm proximally from the base of the first metatarsal along the medial border of the foot. The sheath of the FHL tendon was identified and carefully separated from surrounding tissues using blunt dissection. Special attention was paid to preserving neurovascular structures, particularly the medial plantar nerve and its branches.

An arcuate incision was then made along the posterior medial aspect of the ankle, following the direction of the tarsal tunnel. The posterior tibial neurovascular bundle and tendons were carefully retracted to avoid injury. The point of contrast leakage was identified, and the damaged joint capsule was repaired. Inflammatory tissue was removed, and the FHL tendon sheath was used as a patch to reinforce the repair, ensuring tendon gliding was not impaired.

The arcuate incision followed the posterior border of the medial malleolus, extending 3 cm proximally and 4 cm distally in a curved fashion. The flexor retinaculum was incised, and the tarsal tunnel was opened. The posterior tibial neurovascular bundle was identified and gently retracted anteriorly with a vessel loop. The FHL tendon was then traced proximally to its passage behind the talus, where the contrast leakage point was typically identified. The damaged posterior ankle joint capsule was exposed, and all inflammatory and degenerative tissue was meticulously debrided before repair.

### 2.3 Postoperative management and observation indicators

Postoperative rehabilitation for the great toe began 3 days after surgery under the guidance of a rehabilitation physician. Sutures were removed 2 weeks postoperatively.

**Ankle Joint Imaging**: During surgery, contrast agent was injected into the ankle joint, and fluoroscopy was used to confirm whether the contrast agent leaked through the FHL tendon sheath to the great toe cyst, thereby clarifying the anatomical relationship between the cyst and tendon sheath.

**Functional and Pain Assessment**:

• American Orthopaedic Foot & Ankle Society (AOFAS) score was used to assess great toe function.
• Visual Analog Scale (VAS) was employed to evaluate pain at the great toe, with the following scale: 0 (no pain), 1–3 (mild pain), 4–6 (moderate pain), and 7–10 (severe pain).

### 2.4 Statistical methods

Statistical analysis was conducted using SPSS version 26.0. Quantitative data are presented as means ± standard deviations. Paired sample t-tests were performed to assess changes in pain levels and AOFAS scores for the great toe before and after surgery. A *P*-value of <0.05 was considered statistically significant for group comparisons.

## 3 Results

Intraoperative ankle joint imaging revealed that 14 out of 16 cases (87.5%) were identified as ankle joint-derived cysts, with contrast agent leakage observed through the damaged ankle joint capsule. The leakage extended through the flexor hallucis longus (FHL) tendon sheath to the site of the great toe cyst. Surgical treatment for these cases included excision of the great toe cyst, partial excision of the FHL tendon sheath, and modified repair of the ankle joint capsule. At the final follow-up, no recurrence was observed in these 14 cases.

In the remaining 2 cases, no leakage of the contrast agent from the ankle joint was detected. In these cases, only the great toe cyst was excised. However, one of these patients experienced recurrence, requiring a second surgery, which included arthrodesis of the interphalangeal joint.

In this case of recurrence, the patient presented with recurrent symptoms 5 months after the initial surgery. MRI confirmed the reappearance of the cyst, and repeat ankle arthrography again showed no communication with the ankle joint. This suggested a different pathophysiological mechanism might be responsible for cyst formation in this subset of patients.

**Figure 1:**
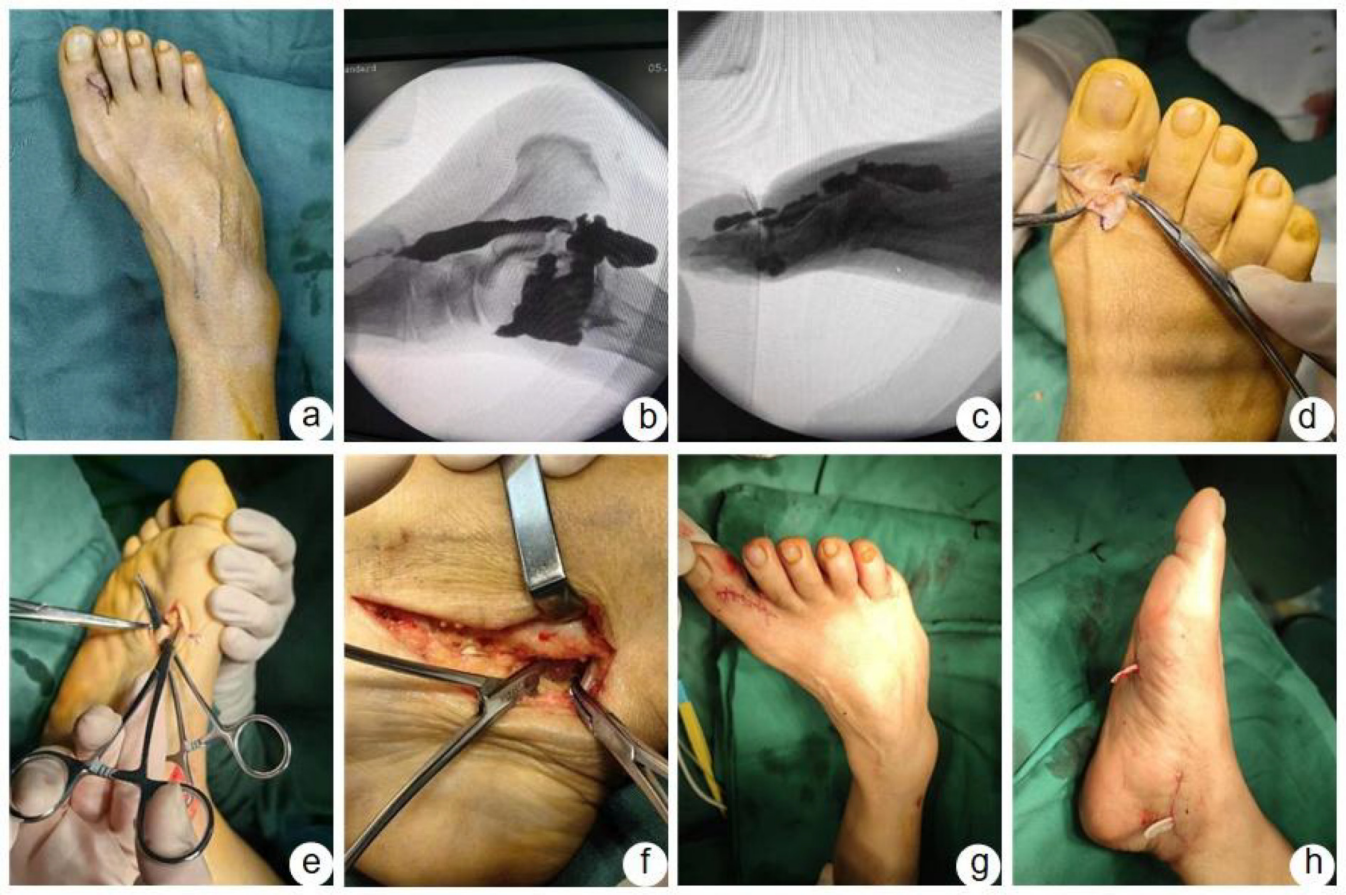
Typical case. Case 6: A 60-year-old female patient presented with a great toe cyst on the right foot, which appeared 1 year ago. After undergoing cyst excision locally, the cyst recurred 7 months later, and was treated with aspiration. The cyst recurred again 2 months later, with pain, prompting a visit for treatment. **(a)** The cyst is visible on the lateral side of the great toe. **(b,c)** Contrast agent leakage from the ankle joint, extending along the flexor hallucis longus tendon sheath to the great toe cyst. **(d)** Exposure and excision of the great toe cyst. **(e)** Exposure of the flexor hallucis longus tendon and partial excision of the tendon sheath. **(f)** Exposure of the damaged ankle joint capsule. **(g,h)** Suturing of the respective incisions.

There were no incidents of vascular, nerve, or tendon injury during the surgery, and all incisions healed without infection. A representative case is shown in Figure 1.

At the final follow-up, both the VAS pain score and AOFAS great toe function score showed significant improvement compared to preoperative levels (*P* < 0.05). The VAS score decreased from a preoperative average of (2.55 ± 1.15) to a postoperative average of (0.83 ± 0.44). The AOFAS great toe function score increased from a preoperative average of 78.55 ± 7.25 to a postoperative average of 93.55 ± 4.90, as shown in Table 3.

**Table 3:** Pain and function scores preoperatively and at the final follow-up (x ± s).

Time	VAS Score (x ± s)	AOFAS Score (x ± s)
Preoperative	2.55 ± 1.15	78.55 ± 7.25
Final Follow-Up	0.83 ± 0.44	93.55 ± 4.90
*t*-value	6.173	−7.516
*P*-value	<0.001	<0.001

## 4 Discussion

Hallux cysts are rare benign masses in the foot, representing approximately 6.6% of all tendon sheath cysts in the foot and ankle region (6). These cysts typically present as a gradually enlarging mass on the lateral side of the great toe. As the cyst enlarges, increased activity may lead to pain, and, in some cases, ulceration and drainage from the cyst's surface, significantly affecting the patient's quality of life (7).

The clinical management of hallux cysts primarily includes conservative and surgical treatments. Conservative methods, such as local compression and aspiration, are simple procedures but exhibit a high recurrence rate, exceeding 50% (8, 9). Surgical options include local cyst excision, cyst excision combined with interphalangeal joint fusion, and arthroscopic excision. Although the recurrence rate is lower after surgery compared to conservative treatment, 12%–42% of patients still experience recurrence (10). The exact cause of recurrence remains unclear; however, it is widely believed to involve mucinous degeneration of the surrounding joint capsule or tendon sheath, potentially resulting from the mucinous degeneration of collagen fibers in the tendons and cell proliferation related to active mucopolysaccharide secretion (11, 12).

Lee et al. (3) defined hallux cysts as “satellite cysts” based on MRI findings. Zhang et al. (4) introduced the concept of ankle-joint-origin hallux cysts, while Wan Dongdong et al. (5) used arthroscopy to observe that the posterior ankle joint capsule was weak and relaxed in patients with ankle-joint-origin cysts, thereby confirming the connection between hallux cysts and the ankle joint via the tendon sheath of the flexor hallucis longus. In this study, all the cases were recurrent hallux cysts, and intraoperative ankle joint contrast imaging revealed that 14 out of 16 cases (87.5%) showed contrast agent leakage through the tendon sheath of the flexor hallucis longus, confirming the existence of ankle-joint-origin hallux cysts.

Anatomical studies have shown that the anterior and posterior ankle joint capsules are relatively weak, with the posterior ligament being the thinnest and weakly connected to the tibia, lower tibiofibular posterior ligament, and talus (13). The posterior collateral ligament of the ankle joint originates from the tibia and fibula and converges on the medial tubercle at the posterior edge of the talus. The tendon sheath of the flexor hallucis longus forms a fibrous tunnel around the tendon, lined by two synovial layers: one lining the inner surface of the tunnel and the other wrapping around the tendon, with both layers connected at the ends of the sheath (13).

Given the unique anatomical relationship between the ankle joint capsule and the tendon sheath of the flexor hallucis longus, the mechanism for recurrent hallux cysts is likely as follows: weight-bearing activity in the ankle joint generates pressure, causing joint fluid to pass through the posterior weak joint capsule into the tendon sheath of the flexor hallucis longus. This fluid accumulates at the tendon and the hallux under pressure, potentially forming a cyst or rupturing if the pressure becomes excessive, especially during repetitive foot and ankle movements. In the cases of our study with positive ankle joint contrast imaging, we observed varying sizes of joint capsule perforations at the junction of the ankle joint and the tendon sheath of the flexor hallucis longus, with joint fluid and contrast agent leakage, which is consistent with the pathophysiological mechanism described in the literature (14).

By using ankle joint contrast imaging, we sought to identify the underlying source of hallux cysts and address recurrence factors at their origin. Zhang Qinglin et al. (9) suggested that unclear preoperative identification of the cyst's origin might lead to postoperative recurrence. Preoperative MRI and ankle joint contrast imaging are crucial for clarifying the origin of hallux cysts. Additionally, since tendons are deep structures adjacent to important blood vessels and nerves, preoperative MRI helps visualize these structures, aiding in the selection of the surgical approach and reducing the risk of unnecessary vascular or nerve injury during surgery (15). While MRI imaging may reveal tendon sheath fluid accumulation and, in some cases, ankle joint effusion, it does not effectively detect joint capsule perforations or confirm cyst origin. Ankle joint contrast imaging, on the other hand, provides real-time observation of the contrast agent's flow, enabling dynamic monitoring of changes during surgery, facilitating identification of the cyst's origin, and allowing for adjustment of the surgical plan in real time. This reduces trauma to the patient and further decreases recurrence factors.

Qu et al. (16) found that extensive excision of the tendon sheath of the flexor hallucis longus effectively treats hallux cysts. Zhang et al. (4) used contrast imaging to identify ankle-joint-origin hallux cysts and adopted a combination of cyst excision, partial excision of the tendon sheath, and ankle joint capsule repair, achieving satisfactory results, consistent with findings in other studies (17). With the development of endoscopic techniques, arthroscopic surgery for hallux cysts, including clearing and opening the joint capsule and arthroscopic tendon sheath excision, has produced good outcomes. However, Wan Dongdong et al. (5) did not reinforce the posterior ankle joint capsule during arthroscopy, but simply opened the joint capsule to allow synovial fluid to drain into the surrounding tissue, avoiding accumulation around the tendon sheath. If the synovial fluid is not absorbed, cyst formation remains a potential cause of recurrence.

More recent studies have validated the approach combining joint capsule repair with cyst excision. Chen et al. (18) demonstrated excellent outcomes using a similar technique for ankle-origin toe tendon sheath cysts, with significantly reduced recurrence rates compared to traditional methods. Their findings support our approach of addressing both the cyst and its originating pathology at the ankle joint level.

In our study, we adopted open surgery, reinforcing the perforated areas of the ankle joint capsule with the tendon sheath of the flexor hallucis longus and thoroughly preventing synovial fluid leakage into the tendon sheath. We also excised part of the tendon sheath, allowing synovial fluid to drain into the surrounding tissue, thus preventing accumulation at the hallux. This approach addresses recurrence factors at their origin. We believe that with accurate diagnosis, a clear understanding of anatomy, careful selection of incisions, and proper protection of nerves and blood vessels during surgery, satisfactory clinical outcomes can be achieved. Compared to arthroscopic surgery, open surgery offers a larger operating space, better visibility, a shorter learning curve, and a lower risk of damaging surrounding blood vessels and nerves, making it more suitable for use in grassroots healthcare settings. In our cases, only one patient experienced recurrence, and upon re-imaging, no connection was found with the ankle joint. After undergoing joint fusion, the patient did not experience further recurrence.

## 5 Conclusion

In conclusion, the use of ankle joint contrast imaging in combination with modified ankle joint capsule repair for the treatment of recurrent hallux cysts effectively identifies the cyst's origin and reduces the likelihood of recurrence by addressing the issue at the source. This approach has shown excellent results.

However, we acknowledge several limitations in our study. The small sample size of 16 patients and the absence of a control group restrict the generalizability of our results. The retrospective design may introduce selection bias, although we included all consecutive patients meeting our criteria during the study period. Additionally, the follow-up period varied between patients (3–24 months), with 75% of patients having ≥12 months of follow-up. Further prospective randomized controlled trials comparing this technique with standard cyst excision without arthrography are necessary to definitively establish the superiority of our approach and validate the clinical effectiveness and recurrence rates.

## Data Availability

The original contributions presented in the study are included in the article/Supplementary Material, further inquiries can be directed to the corresponding author.
